# The Age Friendly Cities and Communities Questionnaire: A Validation Study of the Hebrew Version in Israel

**DOI:** 10.1093/geroni/igaf122.1198

**Published:** 2025-12-31

**Authors:** Liat Ayalon, Shvat Eilat, Jeroen Dikken, Joost van Hoof

**Affiliations:** Bar-Ilan University, Ramat Gan, HaMerkaz, Israel; Bar-Ilan University, Ramat Gan, HaMerkaz, Israel; The Hague University of Applied Sciences, The Hague, Zuid-Holland, Netherlands; The Hague University of Applied Sciences, The Hague, Zuid-Holland, Netherlands

## Abstract

In this presentation, I will report research concerning the reliability and validity of the Hebrew version of the Age Friendly Cities and Communities Questionnaire (AFCCQ). The study was conducted in four Israeli cities, all of which were recognized as age-friendly during the time of the study in June-July 2023, namely: Tel Aviv-Jaffa, Herzliya, Kfar Saba, and Jerusalem. A total of 223 Hebrew speakers over the age of 65 participated in the study. We conducted confirmatory factor analysis to establish structural validity and established the reliability of the measure. Slight dissatisfaction was noted regarding respect and social inclusion across cities. There also was variability across the four cities with Jerusalem being perceived as worse off compared with the other three cities. The findings are discussed considering qualitative interviews with city planners concerning their reflections on the meaning of age friendly cities and communities. Issues of universalism versus age blindness, and age awareness are discussed.

